# Prolonged exposure to neutrophil extracellular traps can induce mitochondrial damage in macrophages and dendritic cells

**DOI:** 10.1186/s40064-015-0932-8

**Published:** 2015-04-02

**Authors:** Luis Donis-Maturano, Luvia E Sánchez-Torres, Arturo Cerbulo-Vázquez, Rommel Chacón-Salinas, Gina S García-Romo, Mariana C Orozco-Uribe, Juan C Yam-Puc, Marco A González-Jiménez, Yuriria L Paredes-Vivas, Juana Calderón-Amador, Sergio Estrada-Parra, Iris Estrada-García, Leopoldo Flores-Romo

**Affiliations:** Department of Cell Biology, Cinvestav-IPN. AV. IPN No 2508, Zacatenco, C.P. 07330 D.F México; Department of Immunology, ENCB-IPN, Col. Santo Tomas, C.P. 11340 D.F Mexico; Department of Cell Biology, INPer., Montes Urales 800, Lomas Virreyes, C.P.11000 D.F México; Department of Nephrology, Leiden University Medical Center, Albinusdreef 2, 2333 ZA Leiden, The Netherlands

**Keywords:** NETs, Dendritic cells, Macrophages, Mitochondrial damage

## Abstract

Neutrophils are one the earliest, crucial innate defenses against innumerable pathogens. Their main microbicidal activities include phagocytosis and degranulation, with many pharmacologically active molecules contributing to inflammation. Recently, a novel antimicrobial mechanism was discovered; the Neutrophil Extracelullar Traps (NETs) formed by extrusion of DNA and associated molecules (histones, elastase, antimicrobial peptides, among others) which trap and kill microorganisms. Since NETs were recently described, research has focused on their induction and microbicidal properties, and recently on disease involvement. However, the functional consequences of NETs interacting with other immune cells, either resident or recruited during early inflammation, have not been assessed.

We therefore investigated the consequences of exposing two major APCs, macrophages (Mfs) and conventional Dendritic Cells (cDCs) to NETs. Our data revealed that at early times (30 min), both Antigen Presenting Cells (APCs) showed induction of important costimulatory molecules (CD80, CD86). Unexpectedly, however, at later times (6 and 24 hours) NETs apparently triggered a cell death process in these APCs by a caspase- and Apoptosis induced factor (AIF)-dependent pathway, suggesting mitochondrial damage. By rhodamine-123 labelling we found that in both APCs, relatively prolonged exposure to NETs or their components importantly decreased the mitochondrial membrane potential. Ultrastructural analysis confirmed mitochondrial alterations in both APCs.

Our results would suggest that early in inflammation, NETs can activate the two main APCs (Mfs and cDCs), but as the process continues, NETs can then initiate apoptosis of these cells through mitochondrial harm. Conceivable, this “late” induction of cell death in these two APCs might start limiting an ongoing inflammatory process to control it.

**Electronic supplementary material:**

The online version of this article (doi:10.1186/s40064-015-0932-8) contains supplementary material, which is available to authorized users.

## Introduction

Polymorfonuclear leukocytes (PMNs) or neutrophils, one of the first effector cells of the innate immune system against infection are the most abundant circulating leukocytes and were discovered by Paul Ehrlich. Neutrophils exhibit numerous molecules to accomplish their functions such as Fc receptors, complement components, antimicrobial peptides, tumour necrosis factor-α, interleukin (IL)-1α, IL-1Ra, IL-12, vascular endothelial growth factor, IL-8, growth-related gene product, macrophage inflammatory protein (MIP)-1α, interferon-γ, among others. PMNs are rapidly recruited to tissues upon injury or infection, where they likely encounter other cells, for instance local and/or recruited dendritic cells and monocytes/macrophages. Until recently neutrophils were thought to perform mainly two essential functions: phagocytosis and degranulation. Upon phagocytosis, PMNs engulf and kill microbes by fusion of phagosomes and lysosomes with granules containing a vast arsenal of antimicrobial molecules (Borregaard and Cowland 1997; Papayannopoulos and Zychlinsky 2009; Segal 2005). During degranulation, neutrophils release to the vicinity of the infectious/inflammatory focus several pharmacologically active molecules, some with antimicrobial properties (Borregaard and Cowland 1997; Fujie et al. 1999).

Recently, Brinkman et al., revealed a novel antimicrobial mechanism for PMNs, whereby activated neutrophils release their nuclear DNA and associated molecules (e.g., histones, elastase, LL-37). This mechanism was first described in human PMNs (Brinkmann et al. 2004), and has since been found in mouse (Ermert et al. 2009), bovine (Lippolis et al. 2006), fish (Palic et al. 2007), and cat neutrophils (Wardini et al. 2010), and in chicken heterophils (Chuammitri et al. 2009). Upon activation, these neutrophil extracellular traps (NETs) are released as a result of a cell death process apparently different from apoptosis or necrosis, which was called Netosis. Netosis depends on the generation of reactive oxygen species (ROS) by NADPH oxidase (Fuchs et al. 2007). NETs have been reported in human appendicitis, experimental shigellosis, preeclampsia (Brinkmann et al. 2004), ulcerative colitis (Savchenko et al. 2011), periodontitis (Vitkov et al. 2009), lupus (Garcia-Romo et al. 2011; Lande et al. 2011), small-vessel vasculitis (Kessenbrock et al. 2009), allergy (Dworski et al. 2011), necrotizing fasciitis (Buchanan et al. 2006), pneumococcal pneumonia (Beiter et al. 2006) and malaria (Baker et al. 2008). NETs are induced by a variety of stimuli such as activated platelets, placental micro-debris, LPS, IL-8, TNF-α, phorbol 12-myristate 13-acetate (PMA), and by multiple microorganisms including bacteria, fungi and protozoan (Brinkmann et al. 2004; Bruns et al. 2010; Gabriel et al. 2010; von Kockritz-Blickwede and Nizet 2009; Ramos-Kichik et al. 2009).

Most studies about NETs are focused either on their experimental induction or the microbial killing, and few about diseases. However, little attention has been paid to the putative interactions between the many active molecules of NETs and the cellular subsets recruited to the inflammatory site. Here, we have identified previously unrecognized features: that NETs can activate macrophages and conventional dendritic cells, but also that they can cause death later on. Our results suggest that APC damage is at the mitochondrial level and that the cell death process triggered seems caspase- and AIF-dependent. Our data suggest that besides the antimicrobial properties, some molecular components of NETs might display -after some time- a deleterious apoptotic impact upon neighboring cells (including resident and arriving/recruited cells), perhaps to start restraining the ongoing inflammatory reaction.

## Materials and methods

### Ethics

This research was performed on healthy competent volunteers in accordance with the Declaration of Helsinki of the world Medical Association, and the Mexican General Health Law regarding research. The ethics committee of the National School of Biological Sciences approved this study (permission number: “Protocolo #CEI-ENCB 011/2013”) and informed written consent was obtained from donors.

### Isolation of human neutrophils and NETs formation

Human blood neutrophils were isolated from healthy donors using Histopaque 1119 and Percoll gradient (Aga et al. 2002). 10^6^ healthy neutrophils/mL of RPMI-1640 medium supplemented with 2% fetal bovine serum (FBS) were stimulated with 100 nM Phorbol 12-Myristate 13-Acetate (PMA) (SIGMA, cat. P-81-39) at 37°C for 4h in 5% CO_2_ atmosphere to optimally induce NETs. After the period for inducing the NETs, the whole cell suspension was centrifuged at 2500 rpm for 5 min and the supernatant was carefully collected (NETs supernatant), while the NETs (in the pellet fraction) were washed twice with RPMI-1640 medium supplemented with 2% FBS to discard the potential effects of –for instance- residual PMA. Thus, this supernatant obtained from the washings of NETs (NETs supernatant) was precisely used as a control (NET sn) to treat the APCs. We checked the NETs sn for the presence of proteins by electrophoresis and by the Bradford method (data not shown). Results shown are the mean +/− Standard Error of Mean of five independent experiments from samples obtained from five different healthy subjects.

### NET fluorescence staining

10^6^ neutrophils/mL of RPMI-1640 medium supplemented with 2% FBS were stuck on 0.001% poly-L-lysine-treated glass coverslips (Sigma Aldrich, St. Louis, MO, USA) and incubated 4h at 37°C in 5% CO_2_ atmosphere in Multiwell Plates (Corning Incorparated. Costar^R^ cat. 3598) with or without stimulus. After incubation, cells were fixed with 4% paraformaldehyde overnight and blocked 2h with 10% normal mouse serum. Cells were then permeabilized with 0.02% Triton X-100 (Polysciences Inc. cat. 4605) in 1 M NaCl and incubated with primary antibodies (mouse anti-human elastase or mouse anti-human histone, both kindly donated by Dr. A. Zychlinsky, Max Planck Institute for Infection Biology, Germany) which were detected with the following secondary antibodies: Alexa Fluor^R^ 488 goat anti-mouse IgG (Molecular Probes, cat. A-11017) and Alexa Fluor^R^ 594 goat anti-mouse IgG (Molecular Probes, cat. A-11020). For DNA detection 4′,6-Diamidino-2-phenylindole-dihydrochloride (DAPI) was used. Specimens were analyzed with a confocal microscope (Olympus BX51TF, Tokyo, Japan).

### Monocyte separation and monocyte-derived macrophages

Peripheral blood mononuclear cells (PBMC) were isolated from buffy coats of healthy donors by Ficoll-Hypaque (Gibco/BRL) density-gradient. CD14^+^ cells were separated by FACS sorting using anti-human CD14-PE (Pharmingen cat. 555398) in the MoFlo™ cytometer (Beckman Coulter). The monocytes obtained were on average 97% pure.

CD14+ monocytes at 10^6^ cells/ml in 24-well plates (Costar, Cambridge, MA) were cultured in RPMI-1640 plus 5% human serum, 1 mM HEPES (GIBCO, cat. 15630), 2 mM L-Glutamine (GIBCO, cat. 25030), 100 UI/mL Penicillin (GIBCO, cat. 15070), 100μg/mL Streptomycin (GIBCO, cat. 15070), 50 μg/mL Gentamicin (GIBCO, cat. 15710) at 37°C in 5% CO_2_ atmosphere. Cultures were fed with fresh medium every 2 days for 6 days.

### Monocyte-derived dendritic cells

CD14+ monocytes at 10^6^ cells/ml in polystyrene 24-well plates (Costar, Cambridge, MA) were cultured in RPMI-1640 plus 5% human serum, 1 mM HEPES (GIBCO, cat. 15630), 2 mM L-Glutamine (GIBCO, cat. 25030), 100 UI/mL Penicillin (GIBCO, cat. 15070), 100 μg/mL Streptomycin (GIBCO, cat. 15070), 50 μg/mL Gentamicin (GIBCO, cat. 15710) at 37°C in 5% CO_2_ atmosphere, supplemented with 1000 U/ml GM-CSF (PreproTech, cat. 300–03) and 200 ng/ml IL-4 (R&D System, cat. 204-IL). Cultures were fed fresh medium and cytokines every 2 days for 6 days.

### Stimulation and staining of dendritic cells and macrophages

To verify monocyte differentiation to macrophages and dendritic cells we used double labeling with CD1a-PE (Santa Cruz Biotechnology, cat. Sc-5265PE) and CD14-APC (Pharmingen, cat. 555399) (Caux et al. 1996). CD14 + CD1a- cells were deemed as monocyte-macrophages while CD14-CD1a + cells were considered DCs. These subpopulations were further screened for the expression of HLA-DR/P/Q (Pharmingen, cat. 3238X), CD80 (Pharmingen, cat. 557227) and CD86 (Pharmingen, cat. 555660).

10^6^ dendritic cells or 10^6^ macrophages were stimulated either with NETs (the pellet fraction) obtained from 10^6^ stimulated PMNs, NET supernatant (obtained from the washing of 10^6^ NETting PMNs) as control, or defined NETs components. Given that we used equal amount of APCs and PMNs to produce the NETs, we describe this as a ratio APCs 1: 1 NETs (we have done also ratios of APCs 1: 2 NETs, shown in Additional file 1: Figure S2). The following NET components were used: Cathepsin G (Sigma Aldrich, Cat. C4428), Elastase (Sigma Aldrich, Cat. E8140), Histone H2A (Sigma Aldrich, Cat. H2042), Myeloperoxidase (Sigma Aldrich, Cat. M6908) and Cathelicidin LL37 (Kindly donated by Dr. L. Cedillo, CINVESTAV, México), during 30 min, 3, 6, 12 and 24 h. Subsequently, cells were treated 5 min with universal blocking reagent (Biogenex, cat HK085-5K) and stained with different combinations of purified antibodies: anti-human HLA DR (Alphachain, cat. M0746), HLA DR/P/Q-FITC (Pharmingen, cat. 32384X), CD80-PE (Pharmingen, cat. 557227) and CD86-APC (Pharmingen, cat. 555660), and Viability Staining Solution: 7-Amino-Actinomycin-D (Pharmingen, cat. 55925).

To evaluate caspase dependent and independent pathways, we used the Fixation/Permeabilization kit (BD cytofix/cytoperm™ cat. 554714) and Rabbit anti-active caspase-3 FITC (Pharmingen, cat. 559341), rabbit anti-human AIF (Abcam, cat. Ab32516), Alexa Fluor^R^ 488 goat anti-rabbit IgG (Molecular Probes, cat. A-11008).

To assess the mitochondrial membrane potential, macrophages or dendritic cells previously stimulated with NETs were harvested, washed with PBS and stained with Rhodamine-123 (SIGMA, cat. R8004) for 30 min at room temperature. After this, cells were washed twice with 2 mL of PBS 1X and analyzed by flow cytometry. As a positive control for the induction of AIF and mitochondrial membrane depolarization, we used carbonyl cyanide m-chlorophenyl hydrazone (CCCP) (Lim et al. 2001; de Graaf et al. 2004) and PBS for caspase-3.

### Transmission electron microscopy

Cell suspensions were fixed 90 min with of 2.5% glutaraldehyde and 4% paraformaldehyde, postfixed 90 min with 1% osmium tetroxide and gradually dehydrated with increasing concentrations of anhydrous ethanol (70, 80, 96 and 100%) and embedded in Epon and absolute alcohol. After polymerization, specimens were cut at 60 nm and contrasted with with uranyl acetate and lead citrate and finally examined by transmission electron microscopy (Zeiss EM10).

### Statistics

Statistics were performed with one-way ANOVA using a Tukey and Bonferroni *t*-test for all multiples pairwise comparisons using GraphPad Prism 5 project.

## Results

### Induction of neutrophil extracellular traps

Human neutrophils isolated from healthy donors were stimulated or not with PMA to induce NETs. The basic content of this extracellular material was analyzed as previously described (Brinkmann et al. 2004) and found that contains DNA (blue fluorescence), elastase (red fluorescence) and histone (green fluorescence), thus confirming the presence of NETs (Additional file 2: Figure S1).

### NETs can activate macrophages and dendritic cells at early time of exposure

**Figure 1:**
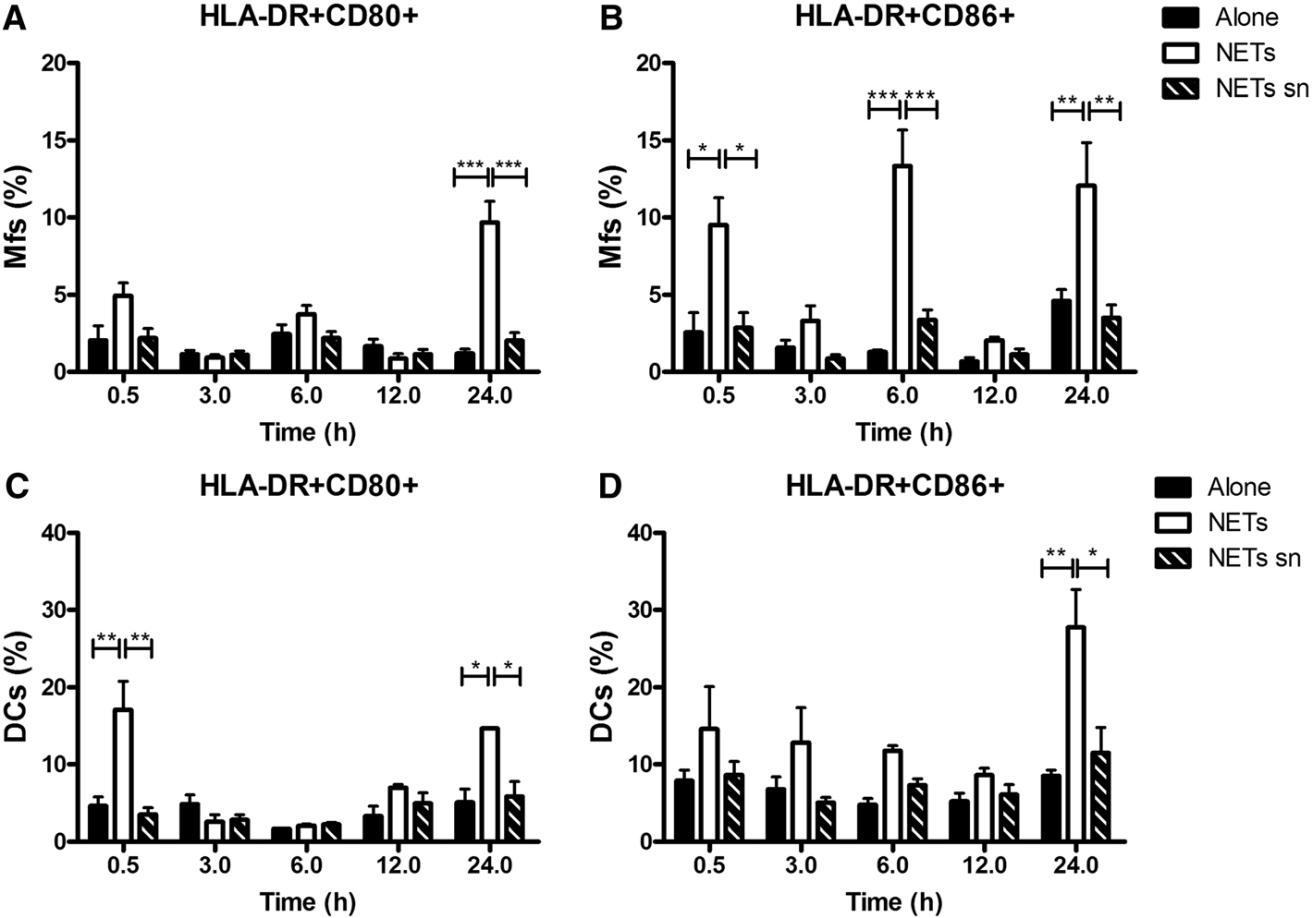
**Activation kinetics of macrophages and dendritic cells upon exposure to NETs from human blood neutrophils.** Percentage of Mfs **(A,B)** or DCs **(C,D)** labeled for HLA-DR, CD80 and CD86 upon incubation only in culture medium alone, with NETs or NETs supernatant as controls. Cells were evaluated at 0.5, 3, 6, 12 and 24h post-stimulation. *P = 0.01; **P = 0.001; ***P < 0.0001, one-way ANOVA. Mfs: Macrophages, DC: Dendritic cells, NETs sn: NETs supernatants.

CD80 (B7-1) and CD86 (B7-2) are crucial costimulatory molecules of antigen presenting cells (APCs), CD86 is constitutively expressed and CD80 is slowly induced and is stable for longer periods than CD86 (Bhatia et al. 2006) therefore we were intrigued by the seemingly bimodal expression of CD86. To assess if NETs would induce APC activation we exposed Mfs or DCs to NETs or to NET supernatants as controls. Mfs and DCs were identified by combining CD1a and CD14 antibodies as described. Exposure of macrophages to NETs increased the HLA-DR + CD80+ macrophages at 24 h (Figure 1A) while HLA-DR + CD86+ macrophages increased at 30 min, 6 and 24 h (Figure 1B). For DCs cultured with NETs we observed increased percentages both in the HLA-DR + CD80+ and HLA-DR + CD86+ populations at 24 h post-stimulation (Figure 1C,D), but the HLA-DR + CD80+ population appeared earlier (30 minutes) (Figure 1C).

### Macrophages and dendritic cells start to die upon prolonged exposure to NETs

**Figure 2:**
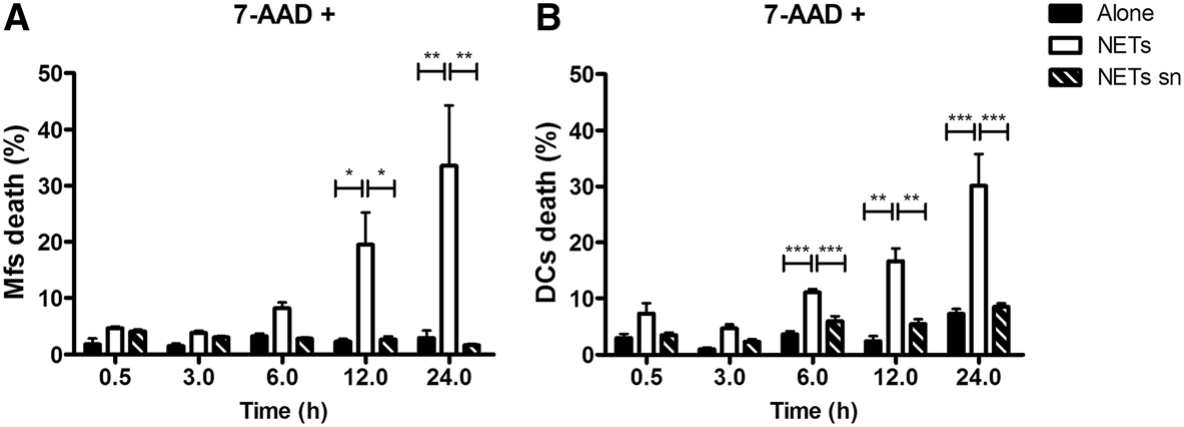
**Death kinetics of macrophages and dendritic cells exposed to NETs from human blood neutrophils.** Percentage of macrophages **(A)** or dendritic cells **(B)** stained with 7-Amino-Actinomycin-D (7AAD) after incubation with culture medium alone, with NETs or NETs supernatants. 7-AAD positive cells represent dead cells and were evaluated at 0.5, 3, 6, 12 and 24h post-stimulation. *P = 0.01; **P = 0.001; ***P < 0.0001, one-way ANOVA. Mfs: Macrophages, DC: Dendritic cells, NETs sn: NETs supernatants.

7-AAD, an actinomycin D analog that binds DNA through GC regions has been used to quantify cell death as the membrane integrity is lost and 7-AAD gains access to DNA (O’Brien and Bolton 1995; Schmid et al. 1992; Schmid et al. 1994). When exposing Mfs or DCs to NETs, we observed increased proportions of dead cells using 7-AAD labelling. This appeared since 6 h until 24h after NETs exposure. We found at 24 h post incubation with NETs 30.58% and 30.16% increase of cell death for macrophages (Figure 2A) and DCs (Figure 2B), respectively.

### Mitochondrial damage in macrophages and dendritic cells exposed to NETs

**Figure 3:**
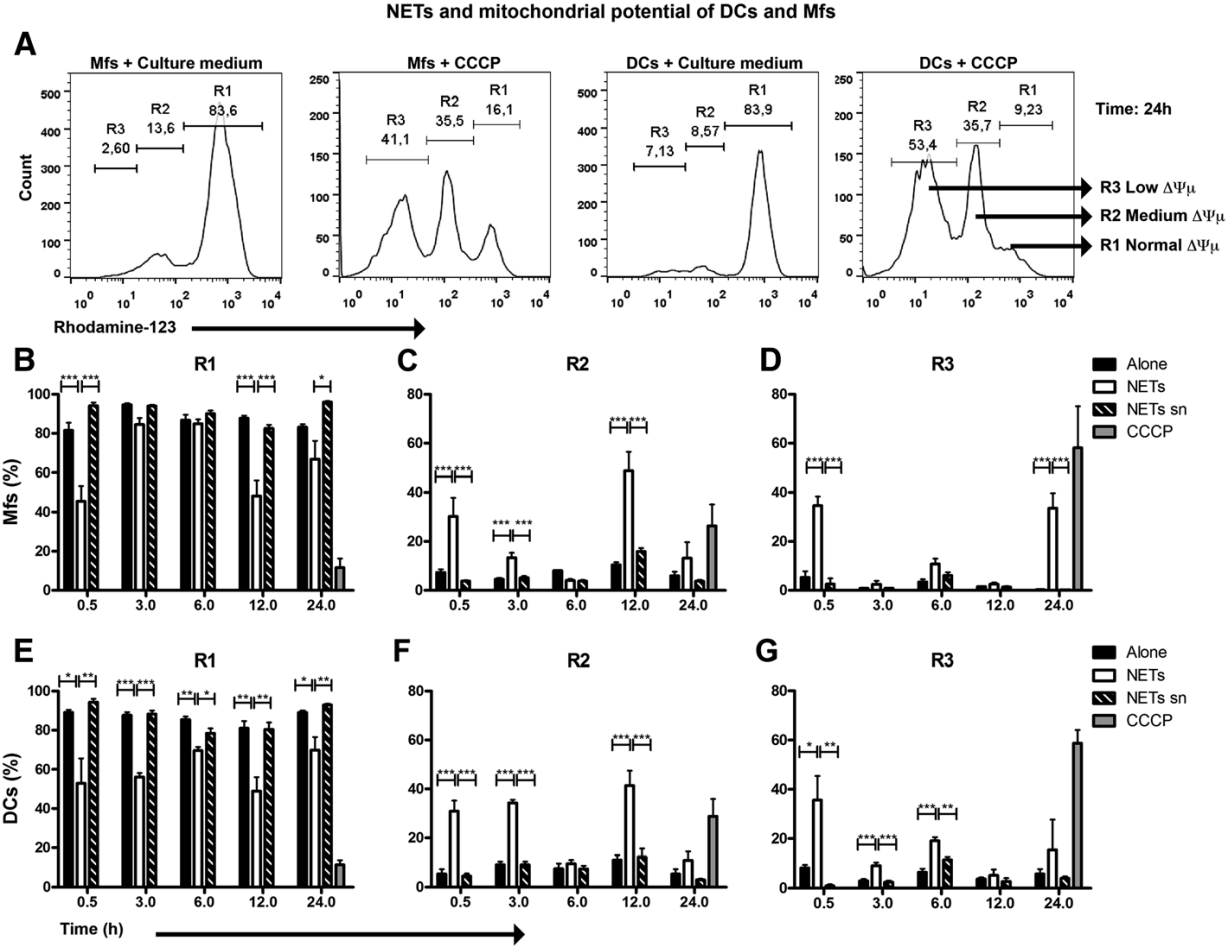
**Analysis of mitochondrial membrane integrity from Mfs and DCs exposed to NETs or NETs supernatants.** Percentage of macrophages **(B-D)** or dendritic cells **(E-G)** incubated with NETs and found in different regions of histograms upon staining with Rhodamine-123. Rhodamine-123 labeling was evaluated at 0.5, 3, 6, 12 and 24h post-incubation with NETs. The histograms shown in **(A)** are examples only to illustrate the regions obtained from two experimental conditions, in this example from Mfs and DCs cultured for 24 hs with medium alone or CCCP (positive control), where R1: normal ∆Ψμ, R2: medium ∆Ψμ, R3: low ∆Ψμ ∆Ψμ: mitochondrial membrane potential, Mfs: Macrophages, DC: Dendritic cells, NETs sn: NETs supernatants, CCCP: Carbonyl cyanide m-chlorophenyl hydrazone. *P = 0.01; **P = 0.001; ***P < 0.0001, one-way ANOVA.

The fluorescent dye rhodamine 123 (Rh123) is used to assess the mitochondrial membrane potential (∆Ψμ. Rh123 stains mitochondria directly, and distributes into the mitochondrial matrix in response to ∆Ψm (Chen et al. 1982; Johnson et al. 1980). When we analyzed Rh123 staining in Mfs and DCs, histograms revealed three clearly distinguished peaks where: R1 corresponds to cells without damage (normal ∆Ψm) in mitochondrial membranes, R2 represents cells with some damage or medium ∆Ψm and R3 corresponds to severe damage in mitochondrial membranes or low ∆Ψm (Figure 3A). We observed that when adding NETs to macrophages, the percentage of live cells (R1) decreased from 30 min (Figure 3B), while that of cells with mitochondrial damage (R2 and R3) increased (Figure 3C,D). The same happened for DCs exposed to NETs (Figure 3E-G).

### Mfs and DCs undergo a caspase- and AIF-dependent cell death after prolonged incubation with NETs

**Figure 4:**
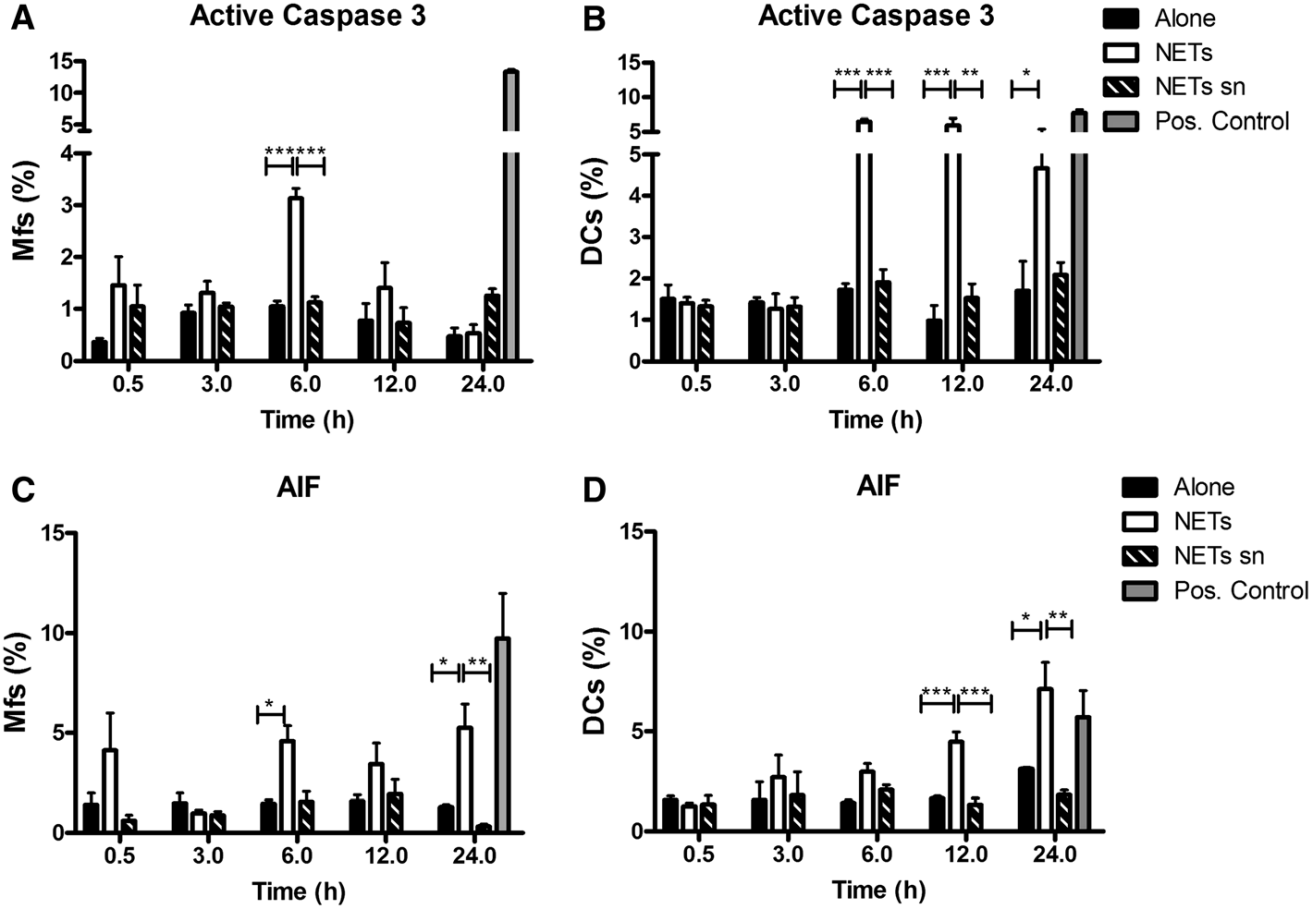
**Kinetics of apoptosis of macrophages and dendritic cells upon exposure to NETs.** Percentage of macrophages **(A,C)** or dendritic cells **(B,D)** incubated only in medium, with NETs, or NETs sn, and labeled for active Caspase-3 **(A,B)** and AIF **(C,D)**. Active Caspase-3 and AIF were evaluated at 0.5, 3, 6, 12 and 24 h post-stimulation with NETs. Positive controls represent Mfs or DCs cultured either in PBS (for Caspase-3) or with CCCP (for AIF) for 24 hours before analysis. Mfs: Macrophages, DC: Dendritic cells, NETs sn: NETs supernatants, CCCP: Carbonyl cyanide m-chlorophenyl hydrazone. *P = 0.01; **P = 0.001; ***P < 0.0001, one-way ANOVA.

To investigate whether apoptosis was involved in the death of macrophages and DCs, both populations were cultured with NETs from human blood neutrophils. Because we observed changes in the mitochondrial membrane with Rho123 staining, we decided to evaluate active caspase-3 and AIF, the latter is a molecule involved in caspase-independent cell death. Results indicated that APCs death was not exclusively through the caspase-3 pathway because these populations (Mfs and DCs) also showed differences in AIF (Figure 4C,D). Compared to control cells we found that in macrophages exposed to NETs, AIF appeared since 30 minutes (Figure 4C). In contrast, AIF increase in DCs was seen until 12 h (Figure 4D).

### NETs caused mitochondrial morphological alterations in both APCs

**Figure 5:**
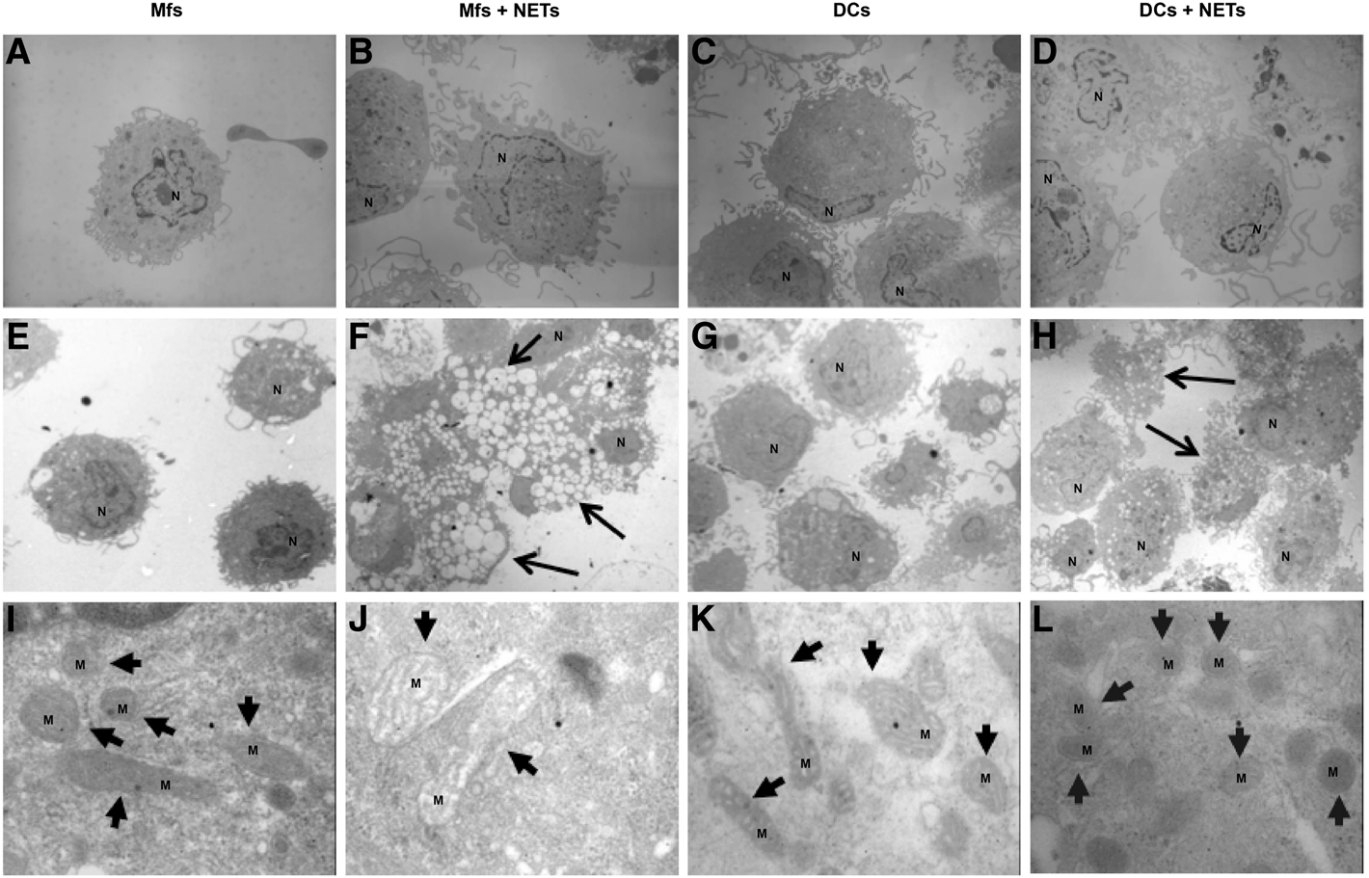
**Ultrastructural analysis by transmission electron microscopy of macrophages and dendritic cells incubated with NETs.** Macrophages **(A,B,E,F,I,J)** and dendritic cells **(C,D,G,H,K,L)** stimulated with NETs **(B,D,F,H,J,L)** or without stimulation **(A,C,E,G,I,K)** at 30 minutes **(A-D)** and 24 h **(E-L)**. Small arrows indicate mitochondria, large arrows indicate vacuolated cells. 3150X **(A-H)** and 50,000X **(I-L)**. M: Mitochondria, N: Nuclei, Mfs: Macrophages, DC: Dendritic cells.

Transmission electron microscopy (TEM) has been an excellent tool in cell death research. To ascertain if NETs induced intracellular changes, we analyzed by TEM the Mfs and DCs cultured with NETs. At 30 minutes, no changes were observed in Mfs and DCs in response to NETs (Figure 5A-D). However, at 24 hours post-stimulation Mfs and DCs exhibited clear cytoplasmic changes (Figure 5F,H) compared with control cells (Figure 5E,G). In NET-stimulated macrophages appeared many cytoplasmic vacuoles as well as ultrastructural mitochondrial changes with loss of lamellipodia (Figure 5F) and mitochondrial crests (Figure 5J). In contrast, DCs treated with NETs had some cytoplasmic vacuoles, little loss of lamellipodia at 24h post-stimulation (Figure 5H) and smaller, circular mitochondria (Figure 5L).

### Macrophages and dendritic cells might die upon exposure to individual NETs components

**Figure 6:**
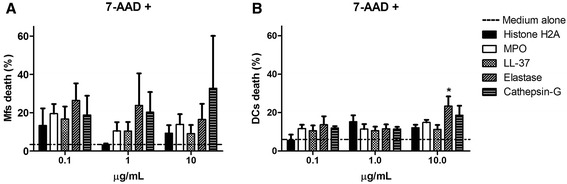
**Effect of various concentrations of individual NETs components on the viability of Mfs and DCs.** Percentage of macrophages **(A)** or dendritic cells **(B)** stained with 7-Amino-Actinomycin-D (7AAD) after incubation with Histone H2A, MPO, LL37, Elastase, Cathepsin G. 7-AAD+ cells represent dead cells and were evaluated at 24 h post-incubation. *P = 0.01. one-way ANOVA. Mfs: Macrophages, DC: Dendritic cells, MPO: Myeloperoxidase, LL-37: Cathelicidin LL-37.

When exposing Mfs and DCs to NETs components (Histone H2A, MPO, LL-37, Elastase, Cathepsin G) for 24 hs, by means of 7AAD we observed a significant proportion of dead cells (Figure 6A,B). For Mfs this was mainly due to elastase and cathepsin G at concentrations of 0.1, 1.0 and 10 μg/mL (Figure 6A), and for DCs was at 10 μg/mL (Figure 6B). Although in Mfs the differences were not significant.

**Figure 7:**
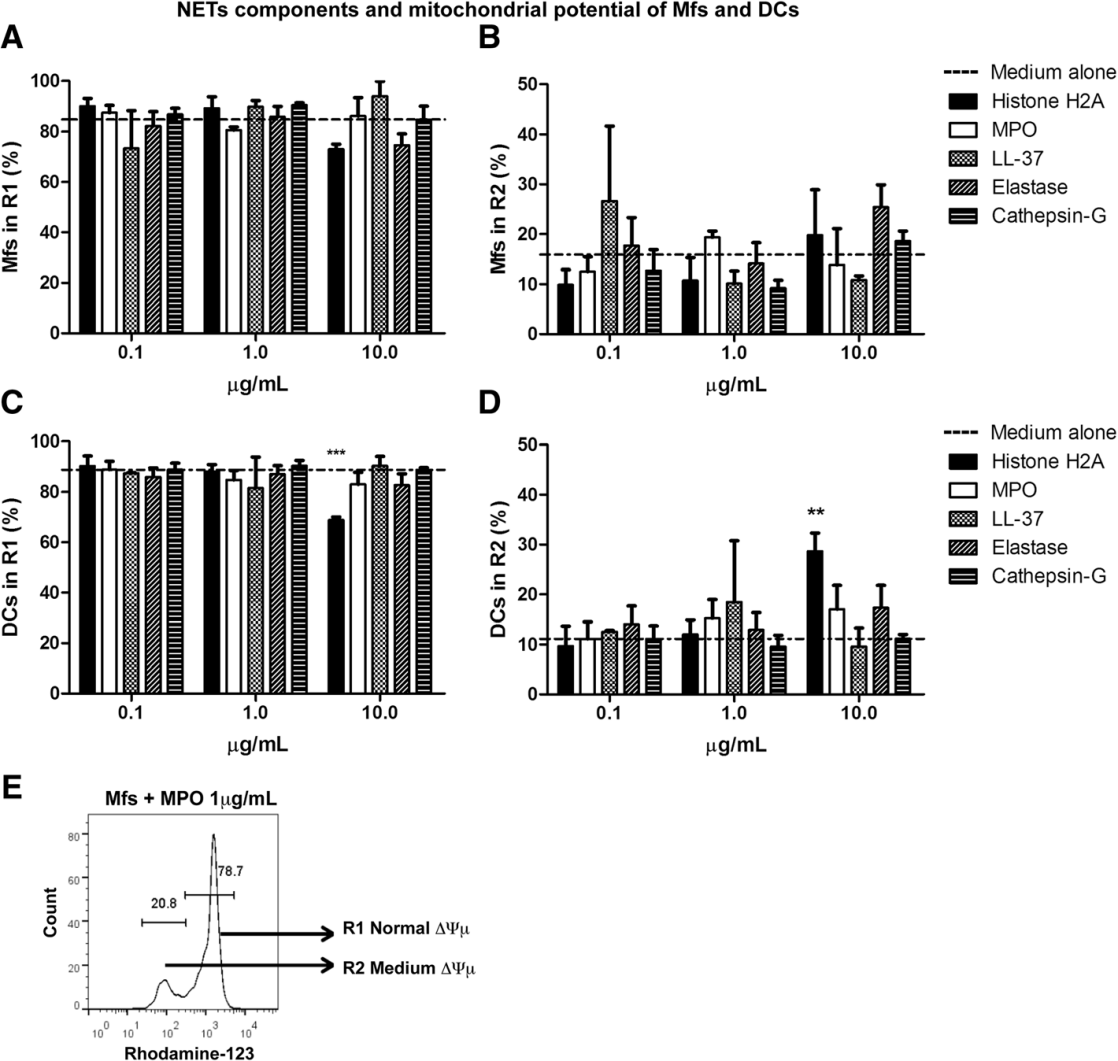
**Analysis of mitochondrial membrane integrity from macrophages and dendritic cells exposed to individual NETs components.** Percentage of macrophages **(A,B)** or dendritic cells **(C,D)** incubated with Histone H2A, MPO, LL-37, Elastase, Cathepsin G and subsequently stained with rhodamine-123. Rhodamine-123 labeling was evaluated at 24 h post-incubation with NETs components. Histogram in **(E)** is one example depicting the regions obtained from one experimental condition, in this case Mfs cultured with medium alone for 24 h, where R1: normal ∆Ψμ, R2: medium ∆Ψμ. ∆Ψμ: mitochondrial membrane potential, Mfs: Macrophages, DC: Dendritic cells, MPO: Myeloperoxidase, LL-37: Cathelicidin LL-37. **P = 0.001; ***P < 0.0001, one-way ANOVA.

Using rhodamine-123 to assess the mitochondrial potential of Mfs and DCs which have been incubated with NETs components, the histograms showed two peaks: R1 (normal ∆Ψm) and R2 (medium ∆Ψm). Interestingly**,** the R3 region (low ∆Ψm), the one corresponding to cells with severe damage in mitochondrial membranes, was lost when APCs were incubated only with individual NETs components (Figure 7E).

When high concentrations of histone H2A (10 μg/ml) were used, the R1region (normal ∆Ψm) decreased significantly, mainly in DCs (Figure 7C). Likewise, the APCs (mainly DCs) with some damage in mitochondrial membranes, found in R2 region or medium ∆Ψm, increased significantly when cells were incubated with histone H2A (Figure 7D),

## Discussion

The leukocytes most abundant in blood are Neutrophils, which integrate the first defensive line against a great variety of microbial pathogens (bacteria, fungi, etc.). Neutrophil microbicidal activities occur through phagocytosis and degranulation (Kennedy and DeLeo 2009). The granules of human neutrophils are recognized for their high content of proteolytic and bactericidal proteins (Welsh and Spitznagel 1971).

PMNs recruited to infected tissue are able to engulf, digest and kill microorganisms as well as the debris ensuing the inflammatory process. However there are multiple components released by neutrophils that can damage neighboring tissues. Moreover professional phagocytes recognize apoptotic cells removing them, thus precluding that aged PMNs discharge their potentially toxic components (Weiss 1989).

In 2004 Brinkmann reported that when neutrophils are activated, they release to the extracellular milieu fibers composed of DNA (apparently the major structural component) and several associated molecules (histones, elastase, LL37, cathepsin G, BPI, lactoferrin, gelatinase, MPO, among others so far identified), these structures were appropriately named “neutrophil extracellular traps” (NETs) because literally trap and kill bacteria, fungi, and protozoa (von Kockritz-Blickwede and Nizet 2009). Furthermore, neutrophils elastase can degrade virulence factors from Gram-negative bacteria (Weinrauch et al. 2002). However, administering elastase into the renal artery of rats has provoked damage with massive proteinuria (Johnson et al. 1988).

While the effects of these various enzymatic components from neutrophil granules upon microbial killing have been long documented, the potential deleterious consequences upon resident and recruited cells during the inflammatory process, have been barely analyzed.

According to our data, depending on the timing of the process, NETs can induce either activation or damage to Mfs and DCs. Clayton described that human monocytes-derived DCs upon ingesting human apoptotic and necrotic neutrophils, increase the expression of CD83 and MHC-II. Of note, if the ingestion of apoptotic neutrophils is high, it can induce down-regulation of the costimulatory molecules CD80, CD86 and CD40 (Clayton et al. 2003). Very recently it was found that NETs efficiently triggered plasmacytoid Dendritic Cell (pDC) activation (Garcia-Romo et al. 2011; Lande et al. 2011). Here we show that upon short exposure, NETs can also induce an increased expression of the costimulatory molecules CD80 and CD86 in macrophages and conventional DCs, both molecules indicative of APC activation. This APC stimulation could be important to develop efficient immune responses subsequent to the neutrophils activities.

However, it has been shown that NETs can also induce damage to neighboring cells. For instance, Gupta showed that activated endothelial cells interacting with transmigrating neutrophils were able not only of inducing NETs, but were also susceptible to NETosis-mediated cell death (Gupta et al. 2010).

We observed similar effects when NETs were added to macrophages or DCs for a relatively “prolonged” period, where the increased proportion of dead cells was noticeable at 6 h and clearly evident at 24 h. When we used rhodamine-123 to assess if there was damage to the mitochondria, we found decreased mitochondrial membrane potential, suggesting an ongoing apoptotic process. This phenomenon was through the caspase-3 and the AIF pathway. Indeed, others have shown that some components of neutrophil granules such as LL37 can induce apoptosis in T lymphocytes, regulatory T cells as well as in airways epithelial cells (Barlow et al. 2006; Mader et al. 2011a; Mader et al. 2011b). On the other hand, elastase and cathepsin G mediated glomerular injury in vivo (Johnson et al. 1988); and cathepsin G has been shown as a critical component sustaining neutrophil-mediated acute tissue pathology and fibrosis after renal ischemia/reperfusion injury (Shimoda et al. 2007). We show here that some NETs components such as histone H2A principally, and to a lesser degree elastase, can cause mitochondrial membrane damage to Mfs and DCs if sufficient time is given. By electron microscopy we observed that in the cultures of APCs exposed to NETs, intracellular changes appeared at the ultrastructural level in mitochondria. This corroborated the results obtained by flow cytometry about mitochondrial membrane damage caused by NETs exposure, suggesting that it might be due to some components of NETs. However it would seem that Mfs are more prone than DCs to extended NETs exposure according to Cytochrome C expression (data not shown).

Our results thus suggest that interaction of NETs or certain NETs components with APCs induced first an activation process upon these cells at early times, but then -if the exposure to NETs continued- a negative effect was seen in cell survival by damage to the mitochondrial membrane, suggesting that some components of NETs might also have, later on, apoptotic effects over these APCs.

An emerging hypothetical scenario is that early in an inflammatory reaction PMNs are attracted to lesional sites, they liberate in situ the NETs with their proinflammatory content to trap and kill potential intruders, thus augmenting the initial inflammation. At this point other cells likely intervening at the inflammatory focus are the two main APCs (Mfs and DCs), which interact with the released material scavenging it while the inflammatory reaction continues. Early after this APCs-NETs interplay is started, the APCs are activated likely getting ready to induce efficient immunity. However, as this interaction continues, apoptotic death is triggered in both APCs, thus the inflammatory reaction starts to decline. As the APCs continue into apoptosis, inflammation could then decline and subside.

## Additional files

Additional file 1: Figure S2. Analysis of mitochondrial membrane integrity from Mfs and DCs exposed either to PMA alone, or to NETs at two different ratios. Percentage of macrophages **(A-D)** or dendritic cells **(E-H)** incubated with medium alone **(A,E)**, 100 nM PMA **(B,F)**, APC 1:1 NETs **(C,G)** and APC 1:2 NETs **(D,H)** for 3 h. Horizontal bars in the histograms indicate the region with normal ∆Ψμ (without damage). Mfs: Macrophages, DC: Dendritic cells. Additional file 2: Figure S1. Neutrophil extracellular traps (NETs). Unstimulated **(A-D)** and PMA-stimulated **(E-H)** blood neutrophils were labeled for DNA (Blue), Elastase (Red) and Histone (Green). DNA staining (DAPI) is shown in **(C,G)**. Immunostaining of neutrophil elastase is shown in **(B,F)**, histone in **(A,E)**, and the merge in **(D,H)**. Stimulated PMNs were washed twice with culture medium containing 5% FBS and the pellets were labeled for elastase, histone and DNA, as indicated, confirming the presence of NETs **(E-L)**. Scale bars: 10 μm.

## Abbreviations

AIF: Apoptosis-inducing factor
APCs: Antigen presenting cells
cDCs: Conventional dendritic cells
Mfs: Macrophages
NETs: Neutrophil extracelullar traps
PMNs: Polymorfonuclear leukocytes
Rh123: Rhodamine 123
∆Ψμ: Mitochondrial membrane potential
