# Supplementary figures and images for: Prolonged exposure to neutrophil extracellular traps can induce mitochondrial damage in macrophages and dendritic cells
Source: Springerplus. 2015 Apr 2;4:161. doi: 10.1186/s40064-015-0932-8 (PMC4392041; doi:10.1186/s40064-015-0932-8)

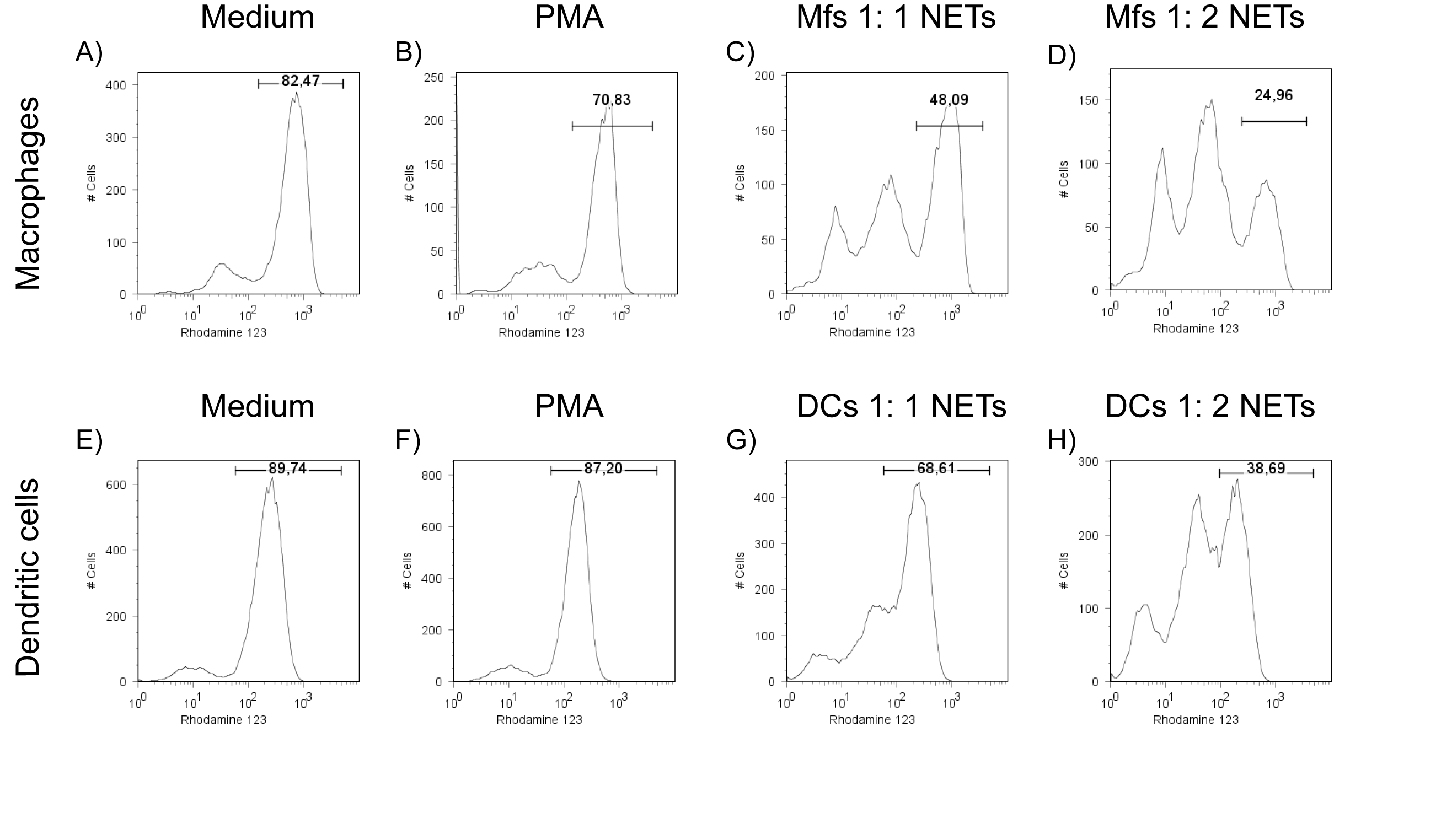

Supplement: Additional file 1: Figure S2. — Analysis of mitochondrial membrane integrity from Mfs and DCs exposed either to PMA alone, or to NETs at two different ratios. Percentage of macrophages (A-D) or dendritic cells (E-H) incubated with medium alone (A,E), 100 nM PMA (B,F), APC 1:1 NETs (C,G) and APC 1:2 NETs (D,H) for 3 h. Horizontal bars in the histograms indicate the region with normal ∆Ψμ (without damage). Mfs: Macrophages, DC: Dendritic cells. [file 40064_2015_932_MOESM1_ESM.tiff]

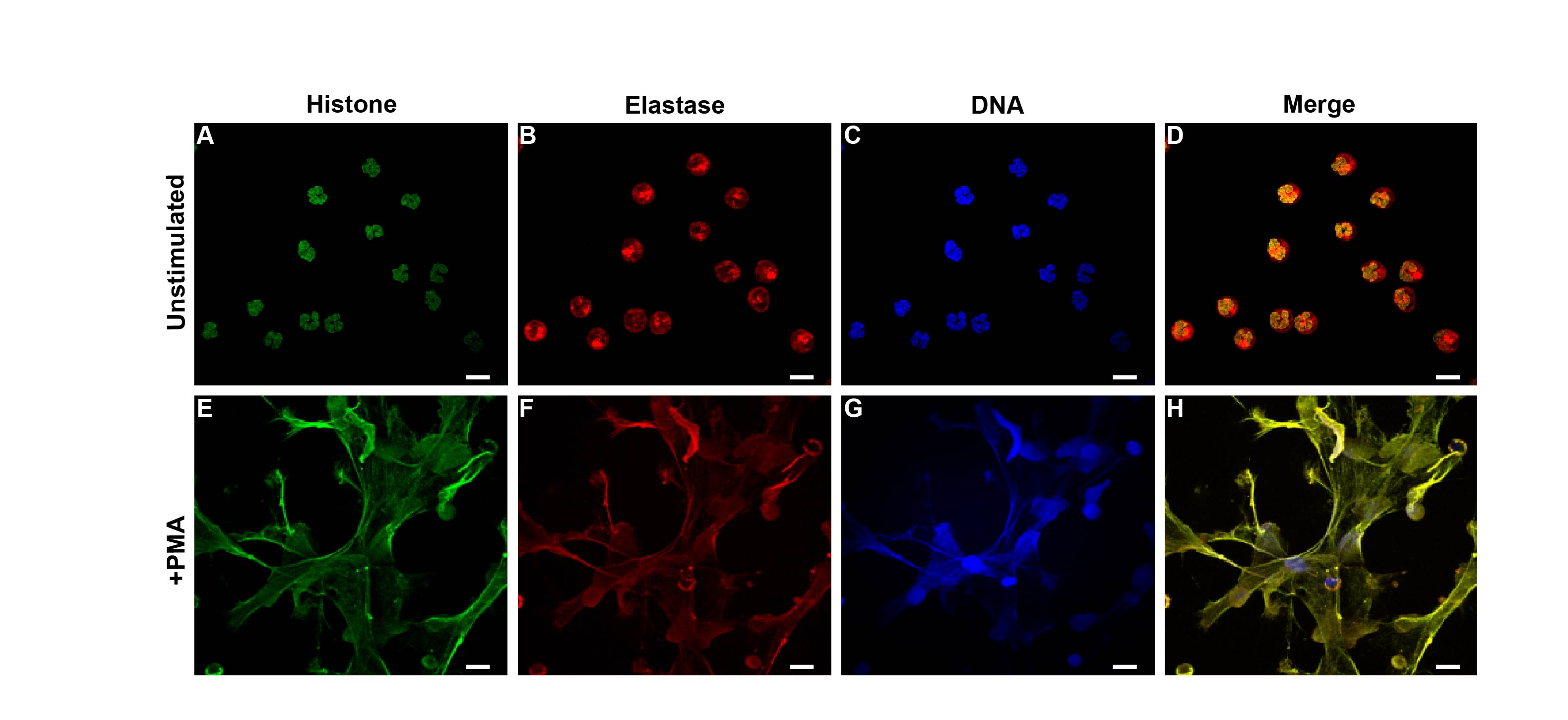

Supplement: Additional file 2: Figure S1. — Neutrophil extracellular traps (NETs). Unstimulated (A-D) and PMA-stimulated (E-H) blood neutrophils were labeled for DNA (Blue), Elastase (Red) and Histone (Green). DNA staining (DAPI) is shown in (C,G). Immunostaining of neutrophil elastase is shown in (B,F), histone in (A,E), and the merge in (D,H). Stimulated PMNs were washed twice with culture medium containing 5% FBS and the pellets were labeled for elastase, histone and DNA, as indicated, confirming the presence of NETs (E-L). Scale bars: 10 μm. [file 40064_2015_932_MOESM2_ESM.jpeg]
